# Internet-delivered unguided psychodynamic vs. cognitive behavior therapy for anxiety and depression symptoms: A large, full factorial randomized controlled trial on treatment length and peer support

**DOI:** 10.1016/j.invent.2026.100980

**Published:** 2026-07-16

**Authors:** Karin Lindqvist, Jakob Mechler, Jón Ingi Hlynsson, Gerhard Andersson, Per Carlbring

**Affiliations:** a Department of Psychology, Stockholm University, Sweden; b The Erica Foundation, Stockholm, Sweden; c Department of Psychology, Uppsala University, Sweden; d School of Psychology, Korea University, Seoul, South Korea; e Department of Behavioural Sciences and Learning, Linköping University, Sweden; f Lusófona University, HEI-Lab: Digital Human-Environment Interaction Labs, Lisboa, Portugal

**Keywords:** Unified Protocol, Affect-focused psychodynamic therapy, Psychodynamic therapy, IPDT, ICBT, internet delivered psychotherapy

## Abstract

**Objective:**

Depressive and anxiety disorders are highly comorbid and widely prevalent, yet a significant treatment gap remains. Scalable, unguided transdiagnostic internet interventions may increase access to care. This study evaluated the efficacy of two distinct unguided transdiagnostic treatments, internet-delivered cognitive behavioral therapy based on Unified Protocol (UP) and internet-delivered affect-focused psychodynamic therapy (IPDT). Furthermore the study examined the impact of treatment duration and assessed the value of peer support.

**Method:**

In a 3 × 2 × 2 factorial design, adults in Sweden with self-reported symptoms of depression and/or anxiety (*N* = 2477) were randomized to one of three conditions: UP, IPDT, or a waitlist control. Participants were also independently randomized to a treatment duration of 8 or 16 weeks, and to receiving access to a peer discussion forum or no forum access. The primary outcomes were symptom severity of depression and anxiety, while quality of life served as a secondary outcome. Data were analyzed using linear mixed models.

**Results:**

Overall, adherence was low, with participants completing fewer than half of the treatment modules on average and high attrition on measurements. At posttreatment, both UP and IPDT resulted in significant reductions in depressive and anxiety symptoms compared to the waitlist control, with small to moderate between-group effect sizes (*d* = 0.38–0.65). Results remained stable during a 24-month follow-up. Both interventions also led to greater improvements in quality of life than the control condition. Head-to-head comparisons revealed no significant differences in primary outcomes between UP and IPDT. Furthermore, extending the treatment duration from 8 to 16 weeks yielded no significant differences in symptom reduction. Access to a peer discussion forum provided no additional benefit.

**Conclusions:**

Unguided transdiagnostic internet interventions can effectively reduce symptoms of depression and anxiety and improve quality of life. Since the 16-week duration provided no added value over the 8-week format, the shorter duration appears sufficient for unguided delivery. However, the low adherence rates suggest that unguided self-help may not be suitable for everyone. The substantial attrition, particularly at follow-up, limits the interpretability of long-term outcomes and highlights the need for strategies to improve engagement in unguided formats.

**Trial registration:**

ClinicalTrials.govNCT05016843

## Highlights

• Largest RCT of internet-delivered psychological treatment to date (*N* = 2477)
• Unguided ICBT and IPDT both outperformed waitlist for depression and anxiety
• No significant differences in efficacy between ICBT and IPDT
• 16-week treatment offered no benefit over 8-week format
• Peer discussion forum did not improve and may have reduced engagement

Anxiety and affective disorders are the two most common groups of psychiatric disorders worldwide, with large health burdens. Despite the high prevalence of these conditions, a discrepancy remains between the need for treatment and the care actually provided. This phenomenon, often referred to as the global mental health gap (Patel et al., 2018), is evident in service utilization rates which can be as low as 2% to 18% for individuals with anxiety or mood disorders (Wang et al., 2007a). There are several evidence-based treatments for affective and anxiety disorders, but there is still a gap where many patients do not receive these. Accessibility factors such as lack of access to psychological treatments and lack of resources within the health care system, in addition to individual factors such as stigma, lack of trust in the health care system and lack of personal resources such as time and transport to get to treatment, are barriers against receiving adequate care (Gulliver et al., 2010). World Health Organization (WHO) reported that the median time from onset of anxiety disorders to treatment in European countries ranges between 16 and 28 years. For mood disorders, the median range is 1 to 3 years (Wang et al., 2007b). The fact that many treatment manuals are diagnosis specific puts a burden on clinicians to learn and be able to competently master treatments for several individual disorders, potentially leading to evidence-based interventions not being offered. Treatment protocols targeting several psychiatric diagnoses, often referred to as transdiagnostic treatments, is one approach intended to increase access to evidence-based treatments. Transdiagnostic psychological treatments have increasingly been recognized for their potential advantages over disorder-specific treatments, particularly in addressing comorbid conditions such as anxiety and depression (Cuijpers et al., 2023). These treatments focus on common underlying factors across different disorders, which can lead to more comprehensive and efficient therapeutic outcomes, in addition to reducing the training burden on therapists, who can develop proficiency in a single framework rather than across multiple disorder-specific manuals.

Several studies have demonstrated that transdiagnostic treatments can be at least as effective as disorder-specific treatments. Meta-analyses have consistently demonstrated that transdiagnostic treatments result in moderate to large effect sizes for both anxiety and depression (Cuijpers et al., 2023; Jiménez-Orenga et al., 2025; Newby et al., 2015). Compared to disorder-specific treatments, data indicate that transdiagnostic protocols lead to similar effects for anxiety with possible superior effects for depression (Newby et al., 2015). While the number of trials has increased substantially in recent years (Cuijpers et al., 2023; Jiménez-Orenga et al., 2025), heterogeneity across studies remain high (Cuijpers et al., 2023; Jiménez-Orenga et al., 2025; Newby et al., 2015). Another approach to increase availability and lower thresholds for seeking and receiving adequate care is the development of internet-delivered interventions (Löchner et al., 2025). A fairly recent systematic review of transdiagnostic internet-delivered treatments found moderate effects compared to passive controls regarding both depression and anxiety symptoms (Kolaas et al., 2024). Another recent review indicated that patients seeking internet-delivered treatment had dramatically longer duration of their depression compared to those seeking care in face-to-face format (10 vs 2.8 years, Aemissegger et al., 2022), suggesting that internet-delivered treatments attract a group of patients unable or unwilling to seek face-to-face treatment.

The Unified Protocol for Transdiagnostic Treatment of Emotional Disorders (UP; Barlow et al., 2017) is a cognitive behavioral treatment focusing on emotions, with five core components targeting temperamental characteristics thought to underly all anxiety and depressive disorders. A recent meta-analysis including children, adolescents and adults reported effect sizes of 0.38 for UP versus active controls and 0.58 versus passive controls. (Carlucci et al., 2021). Another meta-analysis encompassing only adults found within-group effects of *g* = −0.99 for anxiety and *g* = −0.92 for depression, with a global psychopathology effect of *g* = −1.27 (Sakiris and Berle, 2019).

Psychodynamic treatment is transdiagnostic to its nature, even if many modern treatment manuals are tailored to specific diagnoses (Leichsenring and Salzer, 2014). Affect-focused psychodynamic treatment has shown efficacy for a range of disorders (Lilliengren et al., 2025). A transdiagnostic internet-delivered protocol for depressive and anxiety disorders has been evaluated with promising results for both anxiety and depression (Johansson et al., 2012). Recently, another internet-delivered psychodynamic treatment was evaluated showing promising results for adolescent depression (Lindqvist et al., 2020; Mechler et al., 2022; Midgley et al., 2021) as well as slightly adapted for adults with social anxiety disorder (Mechler et al., 2024a). Large effects on comorbid anxiety for patients with depression as well as promising results for patients with social anxiety suggest that this treatment may be suitable to be adapted to a transdiagnostic format.

Even though both the internet-delivered UP and internet-delivered affect-focused psychodynamic therapy (IPDT) are transdiagnostic treatments developed for co-occurring emotional disorders, they approach this shared aim from different theoretical positions. Both UP and IPDT focus on the patient's avoidance of their own emotional experience. However, they do this from different conceptual frameworks. UP construes psychological problems as aversive reactivity to emotions and subsequent attempts to avoid them, and intervenes through a structured, skills-based approach. IPDT construes them instead as unconscious inner emotional conflicts: mixed feelings in key relationships (e.g., anger toward those one also loves, leading to feelings of guilt) generating anxiety and defenses that produce symptoms of depression and anxiety. Therapeutic change does not primarily follow from regulation skills training but from observing, experiencing, and integrating these warded-off, conflicted feelings, often paired with increased insight into the origins of the inner conflict. A direct comparison therefore addresses whether a psychodynamic transdiagnostic treatment can achieve comparable outcomes to an established CBT-based one when digitally delivered. Establishing this is a precondition for the clinically important question of whether particular patient profiles are better suited to one approach than the other.

One common format of internet-delivered treatment is through so called “guided self-help” where the participant receives self-help material which they complete with the guidance and support of a professional (Vernmark et al., 2024). Self-help programmes without guidance have also been evaluated with promising results, where some studies even indicate similar results compared to guided treatment (Dear et al., 2015, Dear et al., 2018). However, meta-analytic results indicate that in general, guided treatment is associated with larger treatment effects (Moshe et al., 2021). Still, if there is a group of patients who benefit from unguided treatment, this is a very cost-effective and flexible format of treatment (Hagberg et al., 2023). Given that the guidance is the most resource-consuming part of the treatment, different levels and forms of guidance have been suggested and evaluated, such as enhanced guidance with chat sessions, guidance from professionals with lower education, patient chat forums, etc. While dose and format are quite well-studied, optimal duration of internet-delivered treatment remains a research gap. In a meta-analysis (Richards and Richardson, 2012), the pooled effect size for studies that had less than eight sessions was significantly higher than studies with eight sessions. However, it is important to note that these studies and interventions may have differed on other key variables such as the type and content of the interventions. The results highlight the potential for future research.

The present trial aimed to evaluate two transdiagnostic unguided treatments with and without peer discussion-forum support and with different lengths of treatment (8 vs 16 weeks). The study had a very low threshold, meaning that anyone screening above cutoff on either the Patient Health Questionnaire (PHQ-9; Kroenke et al., 2001) or the Generalized Anxiety Disorder scale-7 (GAD-7; Spitzer et al., 2006) could be included if not meeting a few exclusion criteria.

## 1 Methods

The study employed a factorial design, where participants were randomized to IPDT, UP or waitlist, for either 8 or 16 weeks, with or without discussion forum. This rendered 12 groups of different combinations of these factors. Randomization was conducted automatically using the Iterapi-platform (Vlaescu et al., 2026) with a 1:1:1:1:1:1:1:1:1:1:1:1 allocation ratio, performed immediately after the collection of baseline data. The study was approved by the Swedish Ethical Review Authority, reference number 2021-00034 l, and prospectively registered at ClinicalTrials (NCT05016843). Primary endpoint was post-treatment, with follow-up at 6, 12, and 24 months.

### 1.1 Recruitment and participants

Participants were recruited nationwide in Sweden between August 2021 and June 2022, through advertisements on the internet. Interested participants could access a study website where they found information regarding the study and the treatment format (i.e., unguided self-help, waitlist, duration and intensity of treatment). A link on the website led to the application. Inclusion criteria were being 18 years or older, able to read and write Swedish, having access to a mobile phone/computer, and rating ≥ 5 on GAD-7 and/or ≥ 10 on PHQ-9. Exclusion criteria were participation in other concurrent psychological treatment, start or dose adjustment of psychopharmacological treatment for anxiety, worry or depression within the last month, severe depression, as indicated by a score of ≥20 on PHQ-9 and suicidality, as indicated by a score of >2 on item 9 on the PHQ-9.

### 1.2 Interventions

Both interventions consisted of text, videos and exercises and were delivered on a secure treatment platform (Vlaescu et al., 2026). Participants automatically gained access to a new treatment module each week, and could work with the material at their convenience. Both treatments were unguided, meaning that there was no feedback on exercises or tracking of progress, but participants could contact the study lead (PC) via the platform if they had questions regarding the study. Automatic reminders were sent when a new module or new questionnaires were assigned. Originally, both treatments consisted of eight modules. For the 16-week treatments, each module was divided in two, resulting in a total of 16 modules. Except from being divided in two, the chapters were identical in the eight- and sixteen-week versions.

#### 1.2.1 Internet-delivered Unified Protocol

Unified Protocol (Barlow et al., 2017; Boisseau et al., 2010) is a transdiagnostic treatment for anxiety and depression. Participants are taught five techniques in order to find new and more helpful ways to react to hindering thoughts and feelings: mindfulness, cognitive flexibility, identification and reduction of behavioral avoidance, increasing willingness to experience physiological sensations and exposure.

#### 1.2.2 Internet-delivered affect-focused psychodynamic therapy

The IPDT treatment is an adapted version of a treatment developed and evaluated for adolescent depression (Mechler et al., 2022; Mechler et al., 2024b), and later adapted for adults with social anxiety (Mechler et al., 2024a). The treatment is based on Malan's triangle of conflict (Malan, 1995), conceptualizing symptoms as results of unconscious emotions, leading to anxiety and defenses. The treatment aims at increasing self-observational capacity as well as helping participants experience and process emotions, regulating anxiety and addressing maladaptive relational patterns associated with inner conflicts.

### 1.3 Discussion forum

Access to a moderated online discussion forum was one of the three randomized factors: half of the participants were given access and half were not. The forum was asynchronous: participants could post their own messages and read other participants' posts. Posts appeared under a username rather than the participant's real name. Its intended function was to provide peer support, on the assumption that this would strengthen engagement and adherence and reduce dropout. Consistent with the unguided format of the treatment, the forum was not used to deliver therapeutic guidance. A clinician from the research team monitored the forum daily, reviewing all posts for safety rather than to provide treatment support, and participants could also flag a post themselves to have it reviewed more quickly. This monitoring followed the trial's general safety procedure, under which a participant showing signs of significant deterioration (for example serious suicidal ideation) was contacted and encouraged to seek appropriate local care.

### 1.4 Measures

All outcome measures were administered via the secure online platform.

Primary outcomes were depression and anxiety. Depression was measured with the PHQ-9 (Hlynsson et al., 2025b; Kroenke et al., 2001). Anxiety was measured with the GAD-7 (Hlynsson and Carlbring, 2024; Spitzer et al., 2006). Both these measures are well-validated, and their brevity make them suitable for weekly assessments. Both primary outcome measures were rated at baseline, weekly during treatment, post-treatment as well as at 6-, 12-, and 24-month follow-up.

Secondary outcome was quality of life, measured with the Brunnsviken Brief Quality of Life scale (BBQ; Hlynsson et al., 2024; Lindner et al., 2016), measured at baseline, post-treatment and 6–12 and 24-month follow-up.

### 1.5 Statistical methods

Analyses were made using Stata 18.0. Statistical analyses of primary outcomes were made using linear mixed models (LMMs). Comparisons were made between predicted end-point scores based on the models. Since there were participants who had completed the baseline measure but no other measures afterwards, in order to conduct a full ITT analyses adjusting for baseline levels of depression/anxiety we followed recommendations by Twisk et al. (2018). Thus, we omitted the main effect of treatment, so the groups were not permitted to differ at baseline (intercept). Models included fixed effects of time and the interaction between group and time, as well as random intercepts and random effects of time. All models were run with time as categorical, not making any assumptions about the functional form of change over time. Covariances were modeled as unstructured, allowing each variance and each pairwise covariance among repeated measurements to be estimated freely.

To assess long-term outcomes up until the 24-month follow-up, we conducted LMMs with time coded as 0 for the post-treatment measurement, 26 for the 6-month follow-up, 52 for the 12-month follow-up and 104 for the 24-month follow-up. Keeping time as a categorical variable led to non-convergence in the models, hence we tested linear, quadratic and cubic time using AIC as a fit index (wherein a reduction in AIC of >2 indicates a meaningfully better fitting model (Burnham and Anderson, 2004)). For all models, quadratic time rendered best model fit. In the context of follow-up, constraining groups to be equal at post-treatment was no longer deemed as justified, as post-treatment outcomes represent a direct consequence of treatment. Accordingly, we did not adjust for baseline in the follow-up analysis.

We also conducted secondary analyses including only participants over cut-off on the respective outcome measures, in order to investigate possible floor effects. The pre-post analysis of BBQ, which was not assessed weekly, was made using ANCOVA following multiple imputation, with the pre-treatment value and treatment group as covariates. 100 imputations were made (Jakobsen et al., 2017). The long-term outcomes on BBQ were assessed using LMMs, adhering to the same principles as described above.

At the end of treatment, participants responded to a few open-ended question about the treatment. Responses were reviewed and summarized descriptively to identify recurring content, providing exploratory context to complement the primary quantitative findings. This was not intended as a formal qualitative analysis but as a brief characterization of participants' experiences.

## 2 Results

### 2.1 Participants

**Fig. 1:**
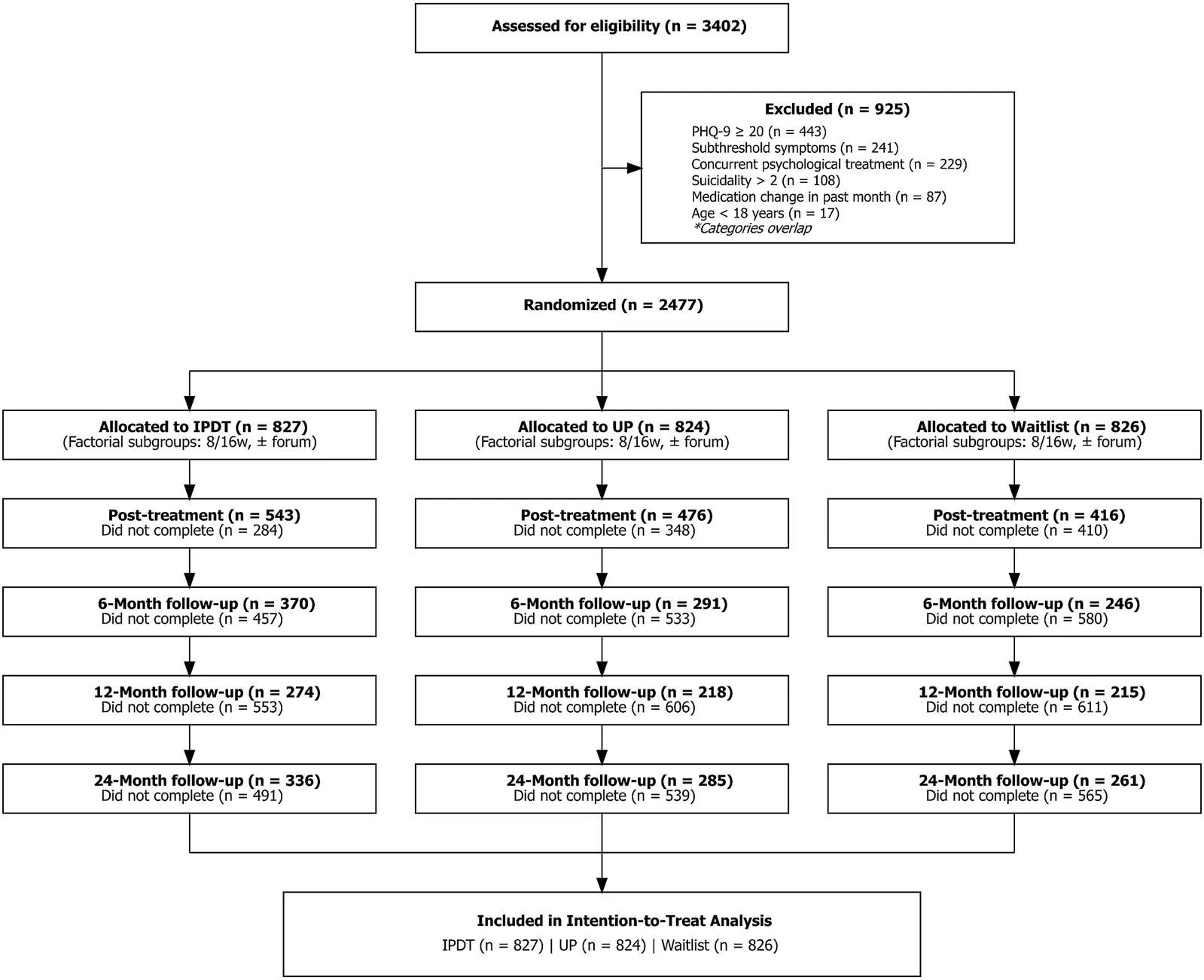
Consort Flowchart

A total of 2477 participants were included in the study (see Fig. 1 for a CONSORT diagram for the study). The mean age was 42.75, (*SD* = 12.46, range 18–86), and 83.4% of the sample were women. Baseline symptom severity was moderate to high, with a mean PHQ-9 score of 11.83 (*SD* = 4.18), and a mean GAD-7 score of 9.18 (*SD =* 4.20). Detailed demographic characteristics by group are presented in Table 1.

**Table 1:** Baseline characteristics for the 12 arms of this 2 × 2 × 3 factorial study, in which participants were randomized to WL (waitlist), IPDT (affect-focused treatment), or UP (unified protocol), each delivered over 8 or 16 weeks, with or without a discussion forum.

	Group (1−12)												Total
	1	2	3	4	5	6	7	8	9	10	11	12	
	(WL 8w no forum)	(WL 8w with forum)	(WL 16 w no forum)	(WL 16w with forum)	(IPDT 8w no forum)	(IPDT 8w with forum)	(IPDT 16w no forum)	(IPDT 16w with forum)	(UP 8 w no forum)	(UP 8w with forum)	(UP 16w no forum)	(UP 16w with forum)	
N	207 (8.4%)	207 (8.4%)	206 (8.3%)	206 (8.3%)	207 (8.4%)	207 (8.4%)	206 (8.3%)	207 (8.4%)	206 (8.3%)	206 (8.3%)	206 (8.3%)	206 (8.3%)	2477 (100.0%)
Age	42.01 (12.55)	44.44 (11.94)	42.37 (12.69)	43.32 (13.06)	42.98 (12.17)	42.14 (11.71)	43.51 (12.08)	42.20 (12.93)	41.91 (11.91)	42.91 (13.27)	41.94 (12.78)	43.28 (12.37)	42.75 (12.46)
Women	169 (81.6%)	175 (84.5%)	179 (86.9%)	186 (90.3%)	180 (87.0%)	165 (79.7%)	170 (82.5%)	166 (80.2%)	161 (78.2%)	175 (85.0%)	172 (83.5%)	167 (81.1%)	2065 (83.4%)
Other	0 (0.0%)	3 (1.4%)	2 (1.0%)	0 (0.0%)	1 (0.5%)	0 (0.0%)	2 (1.0%)	3 (1.4%)	2 (1.0%)	0 (0.0%)	1 (0.5%)	3 (1.5%)	17 (0.7%)
PHQ-9	11.65 (4.15)	11.98 (4.17)	11.29 (4.27)	12.02 (4.16)	12.08 (4.20)	11.07 (4.29)	11.87 (4.37)	12.02 (4.33)	12.64 (3.90)	11.27 (3.90)	12.14 (4.21)	11.90 (4.04)	11.83 (4.18)
GAD-7	9.41 (4.46)	9.94 (4.03)	9.96 (4.22)	9.88 (4.26)	10.12 (4.27)	9.84 (3.75)	9.98 (4.27)	10.43 (4.02)	10.06 (4.37)	9.31 (4.31)	9.86 (4.14)	9.86 (4.27)	9.89 (4.20)

Note: PHQ-9: Patient Health Questionnaire-9, GAD-7: Generalized Anxiety Disorder −7.

### 2.2 Outcomes

#### 2.2.1 Participation

In the 8-week condition, participants in the IPDT group completed 46.5% of the program (*M* = 3.72 of 8 modules, *SD* = 3.08), whereas participants in the UP group completed 37.8% (*M* = 3.02, *SD* = 2.6). In the 16-week condition, the corresponding figures were 44.6% (*M* = 7.14 of 16 modules, *SD* = 6.19) for IPDT and 35.6% (*M* = 5.69, *SD* = 5.39) for UP.

Completion rates differed significantly between groups. In the 8-week condition, 40.3% (*n* = 167) of IPDT participants completed at least five of the eight modules, compared to 27.0% (*n* = 109) of UP participants (χ^2^(1, *N* = 825) = 13.52, *p* < 0.001). Similarly, in the 16-week condition, 36.6% (*n* = 151) of IPDT participants completed at least 10 of the 16 modules, compared to 24.8% (*n* = 102) of UP participants (χ^2^(1, *N* = 826) = 17.89, *p* < 0.001).

#### 2.2.2 Comparison between IPDT and UP

##### 2.2.2.1 Primary outcomes

Across both the 8- and 16-week conditions, both active treatment groups demonstrated significantly larger reductions in depression and anxiety compared to waitlist control. However, there were no significant differences between the two active treatment groups (IPDT vs. UP) on any of the primary outcome measures. Detailed results from the intention-to-treat (ITT) analyses are presented in Table 2.

**Table 2:**
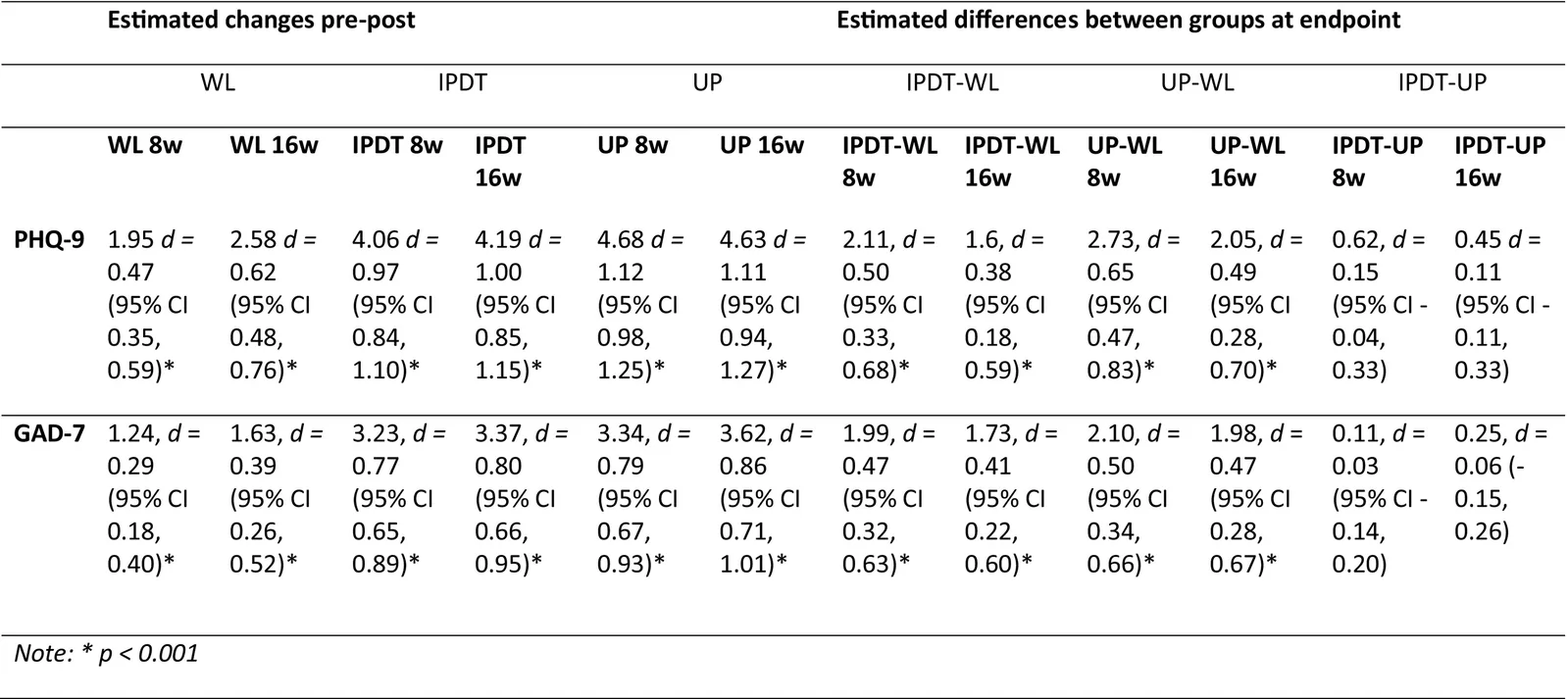
Primary outcomes from ITT analyses.

Note: PHQ-9 = Patient Health Questionnaire-9; GAD-7 = Generalized Anxiety Disorder-7. ^⁎^*p* < 0.001.

For participants over cut-off on the respective measures (10 for PHQ-9 (Kroenke et al., 2001), 10 for GAD-7 (Spitzer et al., 2006)), treatment effects were slightly more pronounced compared to waitlist, but there were no notable differences in the results compared to those for the whole sample. See Table 3 for detailed results.

**Table 3:**
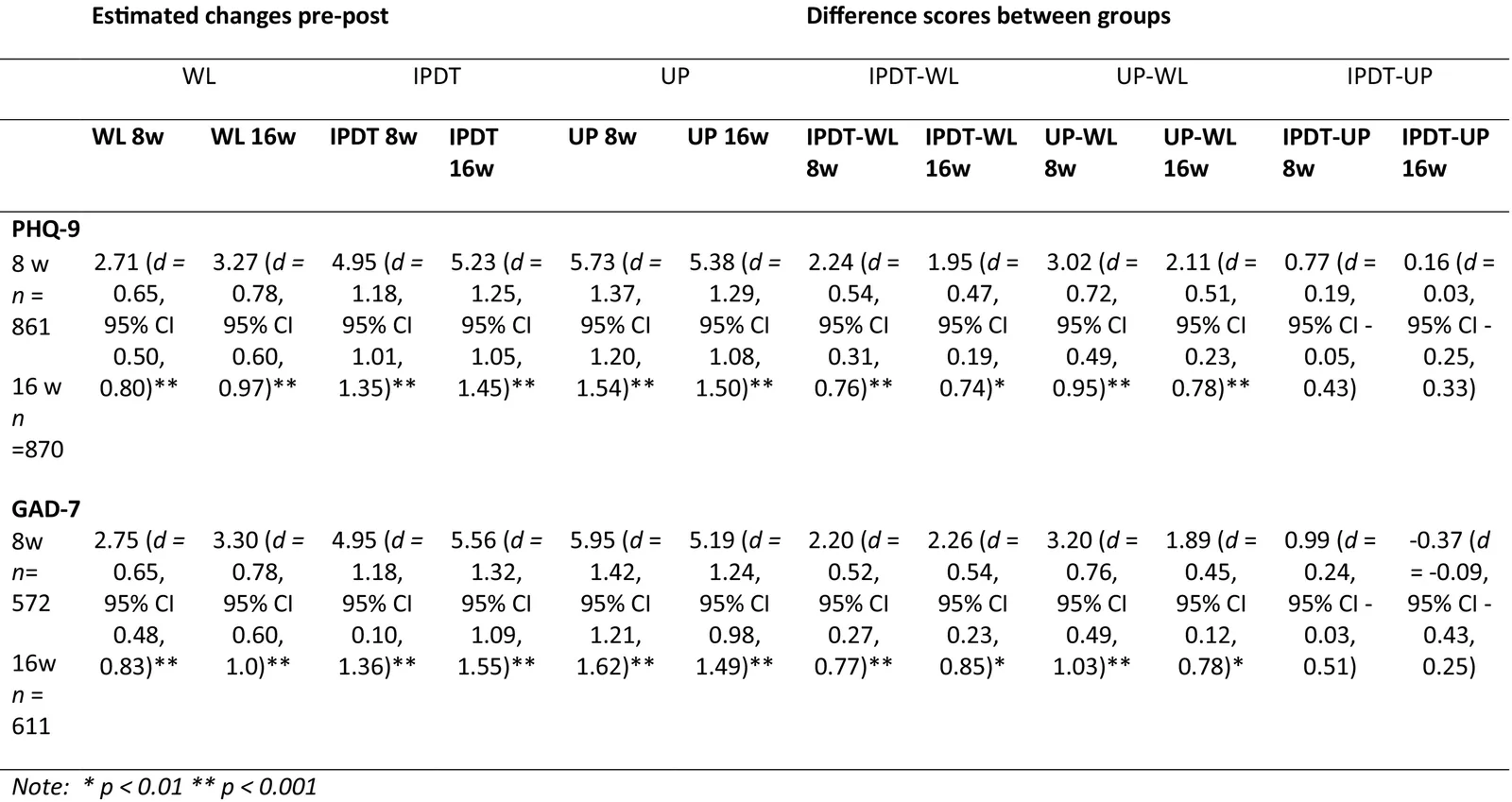
Outcomes from analyses for participants over clinical cut-off at the respective measure.

Note: PHQ-9 = Patient Health Questionnaire-9; GAD-7 = Generalized Anxiety Disorder-7. ^⁎^*p* < 0.01. ^⁎⁎^*p* < 0.001.

##### 2.2.2.2 Quality of life

For BBQ, both treatment groups displayed significantly better scores than the waitlist at post-treatment, with no significant difference between the treatment groups. See Table 4 for detailed results.

**Table 4:** Secondary outcome BBQ, estimated group differences at endpoint.

	Estimated differences between groups at endpoint					
	IPDT-WL		UP-WL		IPDT-UP	
	IPDT-WL 8w	IPDT-WL 16w	UP-WL 8w	UP-WL 16w	IPDT-UP 8w	IPDT-UP 16w
BBQ	6.86, *d* = 0.39 (95% CI 0.26, 0.52)^⁎^	4.64, *d* = 0.27 (95% CI 0.12, 0.41)^⁎^	7.15, *d* = 0.41 (95% CI 0.28, 0.54)^⁎^	6.27, *d* = 0.36 (95% CI 0.21, 0.51)^⁎^	−0.29, *d* = −0.02 (95% CI -0.15, 0.12)	1.63, *d* = 0.09 (95% CI -0.06, 0.25)

*Note:* BBQ: Brunnviken Brief Quality of Life Scale*.*

^⁎^*p* < 0.001.

#### 2.2.3 Follow-up

##### 2.2.3.1 PHQ-9

None of the treatment groups changed significantly during the follow-up period ranging from post-treatment to 24 months (estimated change 0.13, *d* = 0.03 [95% CI -0.12, 0.18] and estimate 0.12, *d =* 0.03 [95% CI -0.13, 0.19] for IPDT and UP respectively). For the 16-week treatment the estimates were − 0.02 *d* = −0.01 [95% CI -0.16, 0.15] for IPDT and 0.55, *d =* 0.13 [95% CI -0.04, 0.30] for UP.

**Fig. 2:**
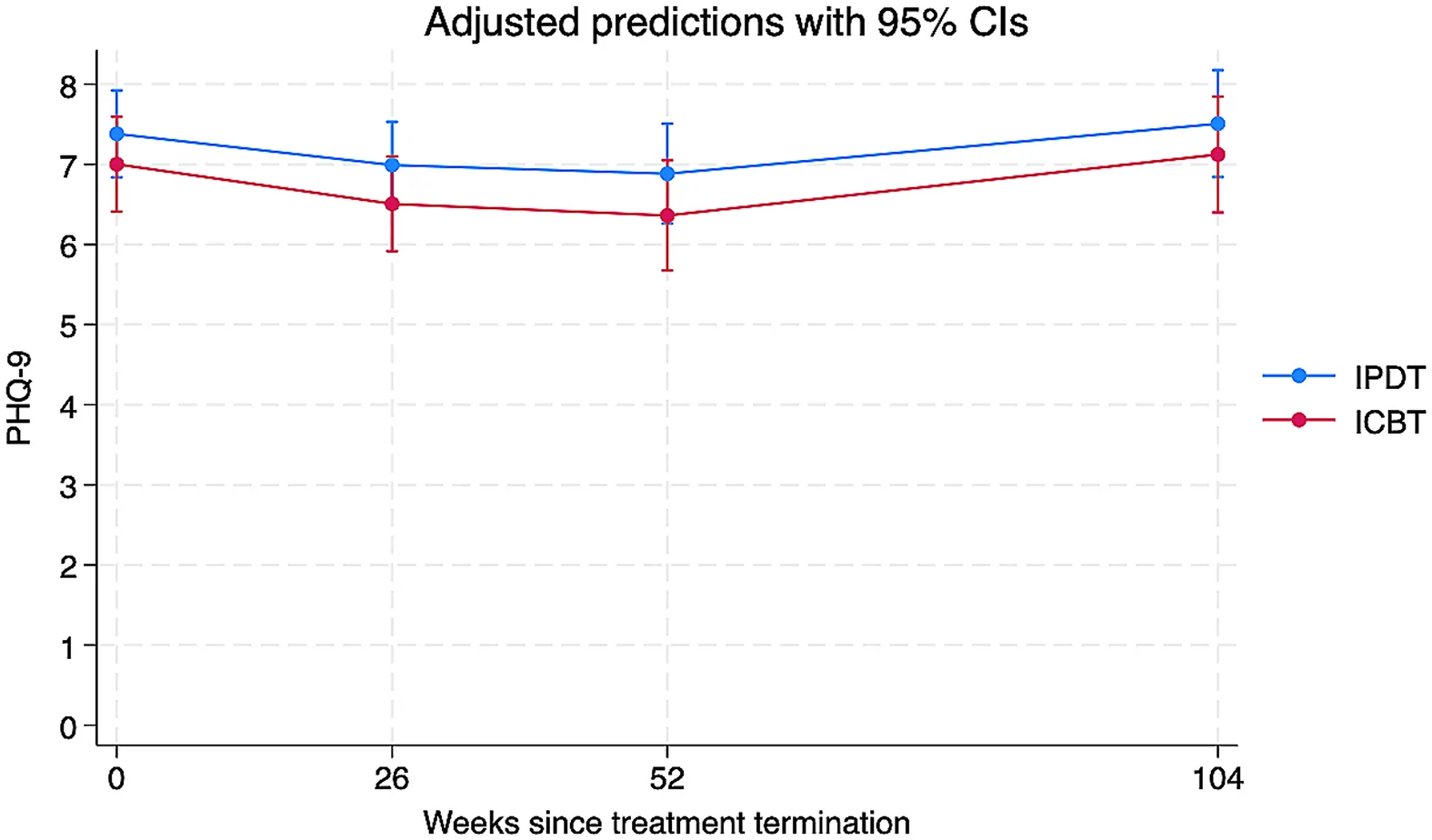
Depression symptoms (PHQ-9) across follow-up in the 8-week condition. PHQ-9 = Patient Health Questionnaire-9.

**Fig. 3:**
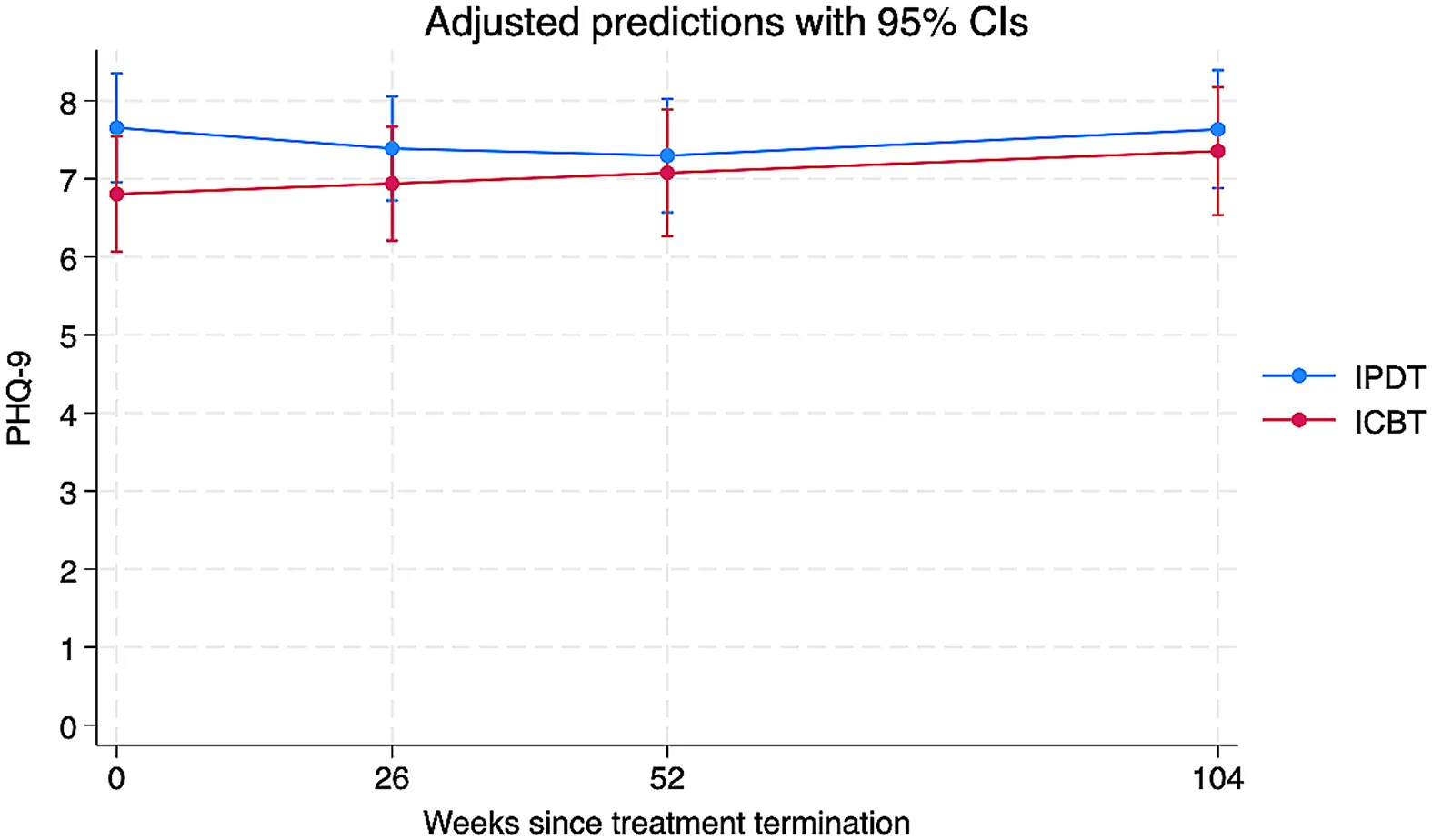
Depression symptoms (PHQ-9) across follow-up in the 16-week condition. PHQ-9 = Patient Health Questionnaire-9.

At the 24-month follow-up, there was no difference between the treatment groups (estimate 0.38, *d* = 0.09, 95% CI -0.33, 0.14 for the 8-week treatment; estimate −0.28, *d* = 0.07, 95% CI -0.33, 0.20 for the 16-week treatment). See Fig. 2, Fig. 3 for illustrations of the trajectories.

##### 2.2.3.2 GAD-7

None of the groups changed significantly during the follow-up period ranging from post-treatment to 24 months. For the 8-week treatment, the estimate was −0.46, *d = −*0.11, 95% CI -0.26, 0.04 in IPDT, and − 0.55, *d* = 0.13, 95% CI -0.29, 0.03) in UP.

**Fig. 4:**
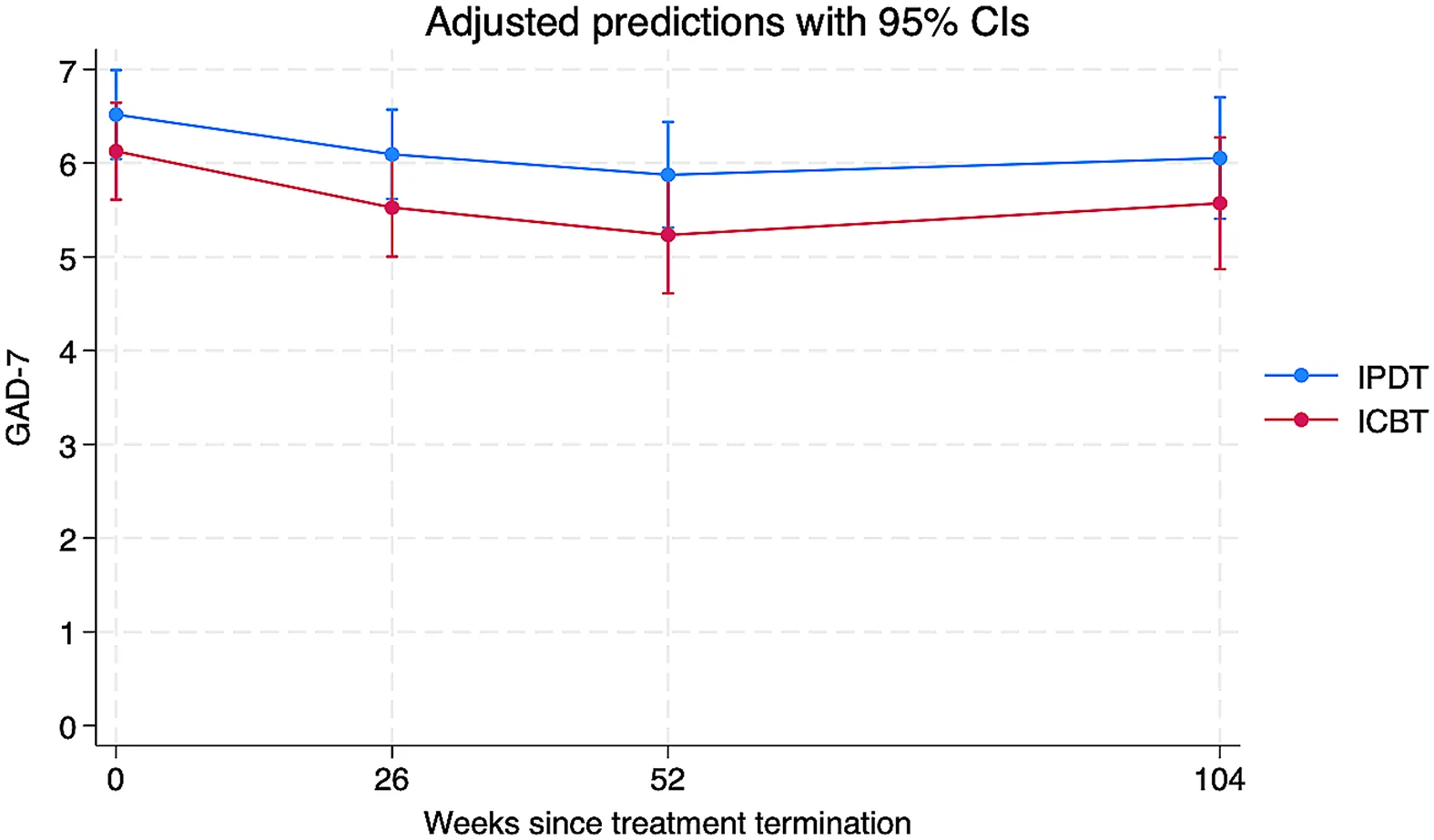
Anxiety symptoms (GAD-7) across follow-up in the 8-week condition. GAD-7 = Generalized Anxiety Disorder-7.

**Fig. 5:**
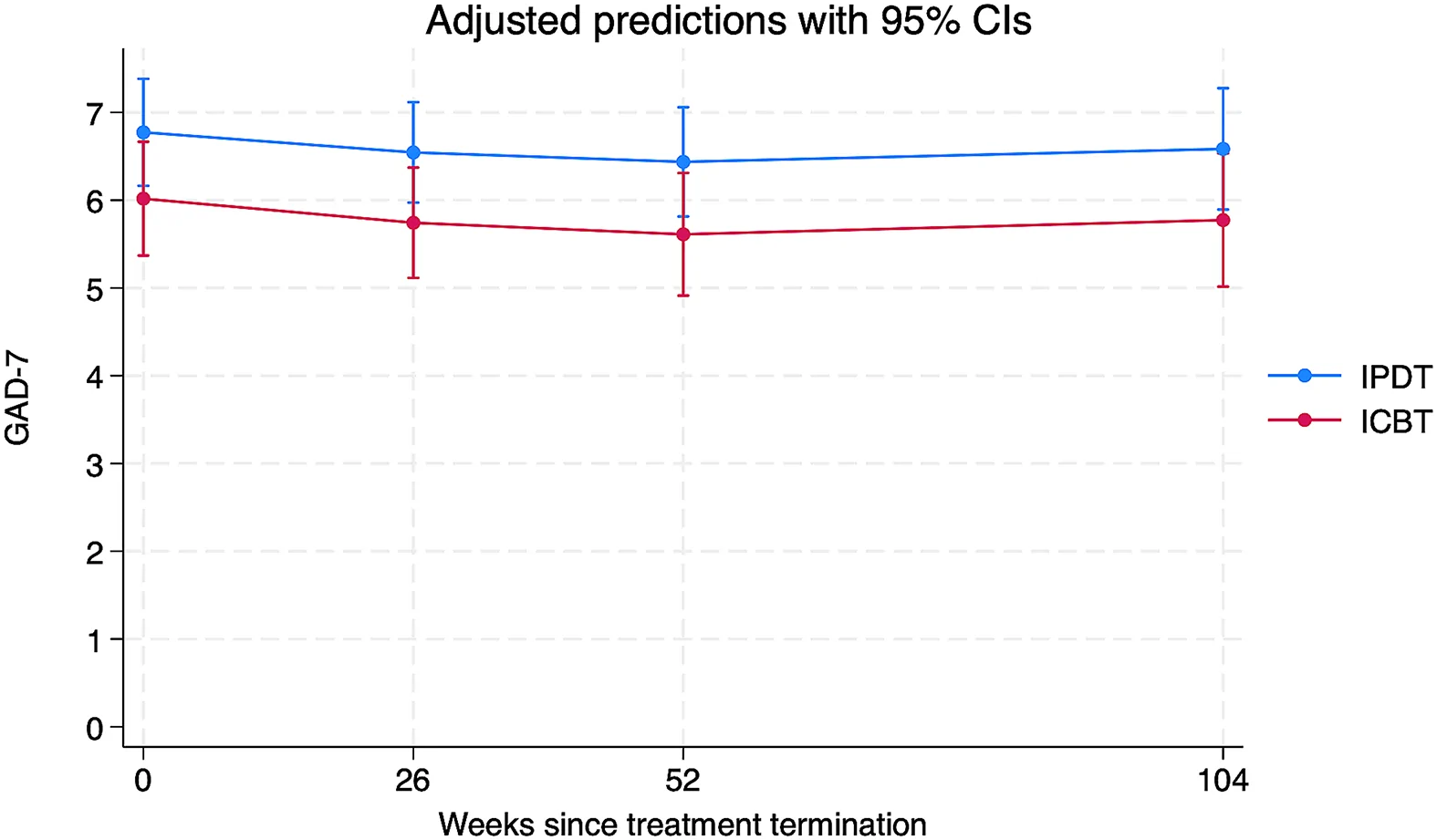
Anxiety symptoms (GAD-7) across follow-up in the 16-week condition. GAD-7 = Generalized Anxiety Disorder-7.

For the 16-week treatment, none of the groups changed significantly during the follow up-period (estimates for IPDT −0.19, *d* = 0.04, 95% CI -0.2, 0.11, for UP -0.24, *d =* 0.06, 95% CI -0.23, 0.11). See Fig. 4, Fig. 5 for illustrations of the trajectories.

At the 24-month follow-up, there was no difference between the treatment groups (estimate −0.48, *d* = −0.11, 95% CI -0.34, 0.11 for the 8-week treatment; estimate −0.81, *d* = −0.19, 95% CI -0.44, 0.05 for the 16-week treatment).

##### 2.2.3.3 BBQ

Both groups improved significantly during the follow up-period. For the 8-week treatment, the estimate was 3.40, *d* = 0.19, 95% CI 0.05, 0.33 in IPDT and 4.01, *d* = 0.23, 95% CI 0.08, 0.38 in UP.

For the 16-week treatment, both groups also improved significantly from post-treatment to the 24-month follow-up. For IPDT, the estimate was 4.28, *d* = 0.25, 95% CI 0.11, 0.38. For UP, the estimate was 4.42, *d* = 0.25, 95% CI 0.11, 0.40.

At the 24-month follow-up there was no difference between the groups in the 8-week treatment. The point estimate was −1.05, *d =* 0.06, 95% CI -0.29, 0.17. In the 16-week treatment, there was a significant difference in favor of UP, point estimate 4.3, *d* = 0.25, 95% CI 0.00, 0.49.

#### 2.2.4 Effects of the forum

In order to explore the effects of forum, we ran a model with forum in interaction with time for the active treatment groups combined. For PHQ-9 there were no significant effects of forum for either the 8 week treatments (estimate −0.70, *d =* −0.17, 95% CI -0.35, 0.01) or the 16 weeks treatments (estimate −0.19, *d =* −0.04, 95% CI -0.27, 0.18).

For GAD-7, there were no significant effects of forum for either 8 the weeks treatments (estimate −0.16, *d* = −0.04, 95% CI -0.20, 0.13) or the 16 weeks treatments (estimate −0.11, *d =* −0.03, 95% CI -0.23, 0.18).

#### 2.2.5 Experiences from the participants

In the post-treatment questionnaire, participants were asked about their experiences of treatment, including questions regarding the length and format of treatment as well as the content of treatment. They were also asked if they would have preferred human guidance. Only 54% responded to the question. Of those responding, 59% responded that they would have preferred human guidance, 26% responded that it didn't matter, and 16% responded that they preferred the self-guided format. In line with this, one of the most common critiques raised in the free-text answers regarded the lack of therapeutic support. Different aspects of this were raised. Some raised feelings of loneliness when facing difficult thoughts and feelings. Others believed they would have been more motivated if they had someone who followed up on their progress and well-being. A third aspect raised was how a therapist might have been able to help personalizing, understanding and reflecting on how the material was relevant to the individual. However, some participants also described it was a relief to not have to “answer to anyone” and that it would have felt more stressful to have a therapist. The other most common negative aspect raised was that the treatment was too time-consuming, to text-heavy or too difficult to fit into everyday life. For some participants, these negative aspects described were prominent in their descriptions of their experiences, whilst for some, this was described in combination with positive aspects such as finding the material interesting, illuminating and the treatment helpful. Positive aspects raised in the free-text responses mainly concerned the treatment content, where the treatment material was described as helpful and, in some cases, even transformative.

## 3 Discussion

This was a large multifactorial trial comparing non-guided internet-delivered unified protocol, affect-focused dynamic treatment and waitlist over eight and 16 weeks, with and without discussion forum, for individuals suffering from depression and/or anxiety. Although suffering from low adherence and large attrition, results indicate that both active treatments were significantly superior to the waitlist control in reducing symptoms of anxiety and depression. However, no significant differences were found between the two treatment modalities. Furthermore, it seems that extending the intervention from 8 to 16 weeks provided no incremental clinical benefit in this unguided format. This aligns with recent findings indicating that extending therapeutic contact, for instance through relapse prevention booster sessions, does not necessarily yield superior long-term outcomes compared to a standard acute intervention (Hlynsson et al., 2025a). This suggests that length of treatment can possibly be adapted due to the needs and preferences of the patient. Similarly, the addition of a peer discussion forum did not enhance outcomes; in fact, trend-level data hinted at a potential negative effect, possibly due to the dilution of therapeutic focus. A further investigation into the activity on the forum is warranted to see how forums are used in this context. This could provide further explanations to why it was not helpful. It is also possible that adding some kind of therapeutic support on the forum, for instance a therapist asking follow-up questions, clarifying misunderstandings or initiating discussions related to the treatment material, in order to facilitate active discussions among participants could enhance the effects of forums while still being time-efficient.

A notable finding was the low treatment adherence, with participants on average completing fewer than half of the assigned modules. This is likely attributable to the low-barrier recruitment strategy. By minimizing inclusion hurdles (e.g., omitting diagnostic interviews), the study likely attracted a highly heterogeneous sample, including individuals with lower motivation or commitment than typically seen in more controlled trials. While this design mirrors the reality of open-access public health interventions, it highlights a trade-off between accessibility and engagement. Consistent with previous meta-analyses (e.g., Moshe et al., 2021), the unguided format likely further contributed to the high attrition rates. Meta-analytic evidence confirms that attrition is systematically elevated in digital interventions delivered without therapist support, in studies using waitlist comparisons, and among online-recruited samples (Jabir et al., 2024; Linardon et al., 2025; Linardon and Fuller-Tyszkiewicz, 2020). In a direct comparison of guided and unguided app-based therapy, dropout was 42.6% in the unguided condition versus 15% in the guided condition (Schittenhelm et al., 2026). Similarly, a large self-guided internet intervention trial for social anxiety (*N* = 2122) found that only 57% of participants provided primary outcome data, with higher dropout in the intervention arm (Powell et al., 2020). Naturalistic data from publicly available unguided programs report that fewer than 15% of users complete all assigned modules (Woolley et al., 2024). The adherence rates in the present trial thus appear characteristic of the unguided, low-threshold format rather than a study-specific anomaly. The fact that 24.8–40.3% of participants finished at least roughly 60% of the modules, and that effect sizes were still significant compared to waitlist, could indicate that unguided treatment can be helpful for a group of patients, but far from all. This is further elucidated by the feedback from participants, where a large group reported having preferred guidance. Efforts should be made to investigate factors determining suitability for these types of self-guided treatments, as this may be an important step in effective resource allocation. Notably, participants in the 16-week conditions remained engaged with the intervention over a substantially longer period than those in the 8-week conditions, whilst still completing a broadly similar proportion of the treatment content. This extended engagement was not related to better outcomes, which were comparable across the two formats. Taken together, these observations suggest that the longer temporal structure lengthened the period over which participants remained involved without a corresponding increase in either content completed or clinical benefit. This has implications for how”dose” is conceptualized in internet-delivered interventions. Dose is often operationalized as the proportion of modules completed, and calendar time in treatment might be considered a further dimension of engagement; yet in the present data neither the additional weeks of involvement nor the format's longer structure conferred measurable advantage. This tentatively suggests that, beyond the engagement afforded by the shorter format, extended duration added little, although the this needs to be further investigated.

Participants in IPDT opened significantly more modules than participants in UP. This could indicate that IPDT was considered lower threshold or more motivating for some participants. At the same time, this was not associated with a larger effect for IPDT. It is also possible that IPDT successfully retained a broader range of patients, including those with more severe or hard-to-treat symptoms who might have otherwise dropped out of UP. This would explain the higher module completion without a corresponding increase in overall effect.

A further question is whether participants who obtained a minimum therapeutic dose differ in outcome, either between treatments or relative to those who did not. We do not analyze this here, because conditioning on attained dose breaks the randomization, and because completion differed substantially between arms for reasons that remain unclear. This means that the completer subgroups may not be directly comparable. Any resulting association would also be open to reverse causation, as improving participants tend to remain in treatment. Establishing a causal effect of therapeutic dose is complicated considering this differential adherence, and we therefore regard it as a question for future work. A related concern applies to the follow-up data. There, only participants who completed at least one post-treatment or follow-up measurement were included, so this subgroup likewise departs from the intent-to-treat sample and is subject to selection. The follow-up results therefore warrant similar caution. At the 24-month follow-up, the 16-week UP group showed significantly higher scores on BBQ than the IPDT group. However, this was not replicated in the 8-week treatment group or when analysing the groups together. Hence, this is a result that should be interpreted tentatively, as it was confined to a single treatment-length subgroup, was absent in the other, and did not persist when the groups were analyzed together.

This is, to our knowledge, the largest sample in a trial of internet-delivered treatment as of yet, meaning that this trial is powered to detect small differences between treatment groups. The inclusion criteria were deliberately wide. The rationale behind this was to investigate whether internet-delivered treatment could be suitable for a wide group of patients suffering from mental health problems. In one way, this could be seen as increasing generalizability, due to the heterogeneity of the group. In another way, the lack of specificity of the group can be seen as a problem, not being a specific diagnostic group, making results slightly more difficult to interpret. However, our sub-sample analyses on participants who scored over cut-off on measures showed similar results as the main analyses, although more pronounced. A strength of the present trial is the inclusion of both a passive waitlist and an active comparison condition (UP vs. IPDT). According to a recently proposed typology for mHealth trials, this design allows for the isolation of intervention effects beyond the passage of time while simultaneously enabling rigorous inferences regarding the relative efficacy of two distinct therapeutic modalities (Goldberg et al., 2023).

Furthermore, this was intended as a minimal-threshold treatment meaning that it was very easy to sign up and participate. Clinically, this increases accessibility and is a strength, but it also leads to obvious problems when analysing results. A major limitation of the present study is the high rate of attrition, particularly at follow-up. While Linear Mixed Models (LMM) handle missing data under the assumption of Missing At Random (MAR), the high rate of missingness in the follow-up data introduces a risk that data may be Not Missing At Random (NMAR). Given this, standard sensitivity analyses (e.g., Last Observation Carried Forward) were deemed likely to yield unreliable estimates. Consequently, the follow-up results should be interpreted with caution and viewed as exploratory, primarily indicating that treatment gains were maintained among those who remained in the study.

Other limitations of the study include not utilizing diagnostic interviews or observer-rated measures. Additionally, reliance on the PHQ-9 constitutes a potential limitation, as recent psychometric evaluations suggest the instrument lacks temporal measurement invariance, implying that score changes over time may not exclusively reflect clinical improvement (Hlynsson et al., 2025b). Furthermore, while the unguided format ensures scalability, it lacks the specific supportive functions, such as personalization, problem-solving barriers, and maintaining momentum, that have been identified as best practices for facilitating engagement in self-help interventions (Shafran et al., 2024).

In conclusion, this is a minimal-threshold unguided study comparing internet-delivered UP, IPDT and waitlist for participants suffering from depression and/or anxiety, with almost no exclusion criteria. Results indicate that participants had very low rates of adherence to treatment but that treatment still, on a group level, was associated with significant effects compared to waitlist. There were no significant differences in primary outcomes between IPDT and UP, and results for 8- and 16-week treatment were very similar. Unguided treatment may be suitable for a group of patients, but this group is probably not representative for the majority. Future research should investigate suitability factors for unguided treatment, as well as for different treatment alternatives. Specifically, future studies should explore if Artificial Intelligence and Large Language Models can be utilized to mimic the supportive functions of a human therapist, such as providing personalized feedback and empathetic responses, to potentially improve adherence and outcomes in scalable digital interventions (Carlbring et al., 2023).

## Declaration of competing interest

None.
